# Prompting Splash Impact on Superamphiphobic Surfaces by Imposing a Viscous Part

**DOI:** 10.1002/advs.201902687

**Published:** 2020-01-10

**Authors:** Fanfei Yu, Shiji Lin, Jinlong Yang, Yue Fan, Dehui Wang, Longquan Chen, Xu Deng

**Affiliations:** ^1^ Institute of Fundamental and Frontier Sciences University of Electronic Science and Technology of China Chengdu 610054 P. R. China; ^2^ School of Physics University of Electronic Science and Technology of China Chengdu 610054 P. R. China

**Keywords:** drop impact, splash, superamphiphobic surfaces, viscous ratios

## Abstract

It is widely acknowledged that splash impact can be suppressed by increasing the viscosity of the impinging drop. In this work, however, by imposing a highly viscous drop to a low‐viscosity drop, it is demonstrated that the splash of the low‐viscosity part of this Janus drop on superamphiphobic surfaces can be significantly promoted. The underlying mechanism is that the viscous stress exerted by the low‐viscosity component drives the viscous component moving in the opposite direction, enhancing the spreading of the low‐viscosity side and thereby its rim instability. The threshold velocity, above which splashing occurs, can be tuned by varying the viscosity ratio of the Janus drop. Moreover, the impact of the Janus drop can be employed to verify the mechanism of splash.

## Graphical abstract

When a single‐phase water drop impacts on a superamphiphobic surface, it symmetrically spreads and completely bounces off the surface. Surprisingly, by imposing a highly viscous drop to the water drop, the resulted Janus drop exhibits an asymmetric spreading and retracting phase, and the splashing of the water part is significantly promoted compared to the single‐phase water drop impact.

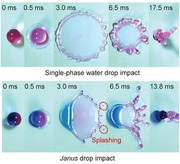

The impact of liquid drops on solid surfaces is the most prevalent phenomenon of capillarity that has been extensively investigated over the past century,^1^ yet this transient free‐surface flow has not been characterized in finer detail, until recently, due to the development of fast‐imaging techniques.^2^ Depending on the impact conditions, the fate of an impinging droplet could be a regular deposition,^3^ a ballistic rebound,^4^ a pancake rebound,^5^ a gyrate rebound,^6^ or a splashing.^7^ Among them, the splash impact, i.e., break up and ejection of small droplets, is not only an intriguing scientific problem spanning a broad range of length and time scales, but also a key technical issue involving in many industrial processes.[qv: 7a] In some applications, such as ink‐jet printing,^8^ and pesticide deposition,^9^ splash is undesirable for controlling droplet deposition and thus it needs to be eliminated; while for others, such as spray cooling^10^ and internal combustion engines,^11^ splashing is beneficial. Accordingly, it is significantly meaningful to understand the physical mechanisms of splashing and find parameters or strategies to control its occurrence.

Splashing has been generally classified into two distinct categories with respect to experimental observations: corona splash and prompt splash.^3^ While corona splash frequently occurs on smooth surfaces,^7^, ^12^ where a symmetric corona first forms, then expands and eventually breaks into small droplets, the prompt splash releases small droplets on rough surfaces from the tip of lamella at the contact line.[qv: 7a,13] The threshold velocity, above which the splash can be produced, was found to be set up by the characteristic physical parameters of the single‐14 or multi‐component^15^ impinging drop, roughness and mechanical property of the target surfaces,^12^, ^16^ the ambient pressure,[qv: 7b] and also the surface wettability.^17^ On the other hand, theoretical analyses^18^ and numerical simulations^19^ have been employed to explore the generation mechanism of splashing. A number of elegant models, which concern the early‐time inertial dynamics,[qv: 16b,20] the Kelvin–Helmholtz (KH) instability,^21^ Rayleigh–Taylor (RT) instability,[qv: 19a,22] the aerodynamics of the entrapped gas layer,[qv: 18a] and the lamella lifting mechanism,^23^ have been proposed. However, these models can only capture part of the splashing phenomenon and apply to some specific systems. Despite the bitter debate on the origin of splashing, it has been widely accepted that increasing the viscosity of the working fluid can effectively delay or even suppress the splash due to the highly viscous dissipation.[qv: 16a,23a] In this work, however, we explore a new intuitive topic, namely, how to trigger splashing by increasing the viscosity of the impinging drop.

We demonstrate a novel interfacial phenomenon in which the splash of a low‐viscosity drop can be significantly prompted by adding a rather viscous component. Systematical experiments demonstrate that the splash threshold velocity decreases with increasing viscosity ratio. Further analyses suggest that the viscous shear force exerted by the highly viscous component enhances the spreading of low‐viscosity component and thus the splashing phenomenon. Our finding provides a novel strategy to control drop splash.

In our experiments, the impinging Janus drop was created by adding a glycerin drop to a water drop (**Figure** **1**a; Video S1, Supporting Information). Note that, while the Janus drop spins slowly when it falls downward, the rotation speed in the horizontal component can be ignored compared with the vertical impact speed (Figure S1, Supporting Information). For consistency, we only study the impact dynamics when the interface of the Janus drop is perpendicular to the superamphiphobic surface at the contact moment (with an angle error ≤ 5°, Figure S2, Supporting Information). To simplify the discussion, we employ the viscosity ratio *µ*_h_/*µ*_l_ to denote the Janus drop, where *µ*_h_ and *µ*_l_ represent the viscosity of the highly viscous part and low‐viscosity part, respectively. *µ*_h_ was varied using water–glycerin mixtures at different ratios. We point out that the mixing of water and diverse glycerin–water mixtures can be neglected during the impact as the diffusion of glycerin into water during impact is only several tens of micrometers (see the Experimental Section; Figure S3, Supporting Information), which is at least 100 times shorter than the millimeter‐sized droplets. It is noted that we only focus on the effect of the viscosity ratio of the Janus drop on splash behaviors in this work. Thus, the water‐ glycerin/water mixtures were chosen since they have similar surface tensions, which allows for the formation of spherical drops with two parts of different viscosities. The impinging drops were released from different heights above the horizontally placed superamphiphobic surface^24^ (see the Experimental Section; Figure S4, Supporting Information). The drop impact processes were recorded using two high‐speed cameras from the top and side views concurrently. For visualization purposes, Janus drops were colored by two food dyes (mass fraction ≈ 0.5‰).

**Figure 1:**
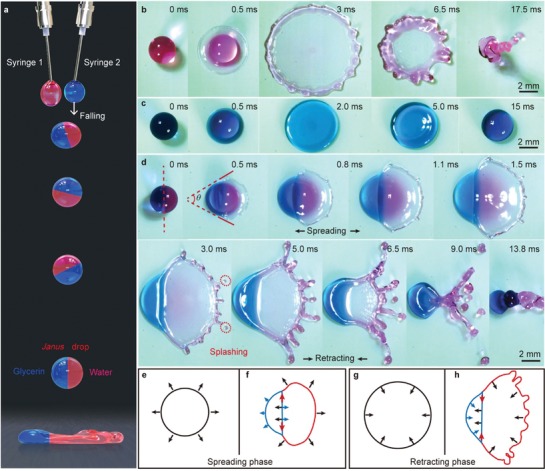
Concept and prompt splashing of Janus drop. a) Schematic of experimental setup. The Janus drop is generated by gently contacting water and glycerin drop. Selected high‐speed images show the dynamics of drops (*D* ≈ 3.1 mm) impacting on superamphiphobic surfaces at *v* ≈ 2.2 m s^−1^. b) The water drop (*µ* ≈ 1 mPa · s). c) The 95 wt% glycerin drop (*µ* ≈ 545 mPa · s). d) The Janus drop (*µ*_h_/*µ*_l_ ≈ 545) contains 95 wt% glycerin (blue part) and water (red part), exhibiting prompt splash. Schematic of the typical phases of the e,g) single‐phase drops and f,h) Janus drops during drop impacting.

The representative snapshots of impinging single‐phase drops and a Janus drop on the superamphiphobic surface at an impact velocity *v* ≈ 2.2 m s^−1^ are shown in Figure 1b–d. Both the pure water (*R*_w_ ≈ 1.55 mm, *µ* ≈ 1 mPa · s) and glycerin drops (*R*_g_ ≈ 1.55 mm, *µ* ≈ 545 mPa · s) show nearly symmetric dynamics throughout the impact process (Figure 1b,c; Video S2 and Figure S5a,b, Supporting Information). In contrast, an asymmetric shell‐like shape was observed for the impinging Janus drop $\left(\mu_{\text{h}}\text{/}\mu_{\text{l}}\approx 545\right)$ due to the different spreading rates of glycerin and water (Figure 1d; Video S2 and Figure S5c, Supporting Information). Upon impact, the glycerin side of the Janus drop spreads out smoothly in a near‐semicircle manner for a few milliseconds, while the water side fans out promptly with an open angle (θ) of ≈90°. Even more remarkably, multiundulations are formed on the rim of the water part of the Janus drop, resulting in impending splash. Subsequently, micro‐sized daughter droplets (red circle) are ejected at the wetting front of the water part (Figure 1d). However, no splash behaviors were observed for a pure water drop though the impact velocity is the same (Figure 1b). The above comparative experiment suggests that if a low‐viscosity drop is partially substituted by a highly viscous component, its fate during impact would be influenced by the asymmetry spreading dynamics, as shown schematically in Figure 1e–h.

To further explore the effect of the viscosity difference on the asymmetric impact dynamics of the Janus drop, we performed the experiments with the viscosity ratio *µ*_h_/*µ*_l_ ranging from 112 to 1491. Note that further decreasing *µ*_h_/*µ*_l_ would obtain an unclear water/glycerin interface since the mixing between two components becomes stronger. Similar asymmetric impacting dynamics at *v* ≈ 2.2 m s^−1^ are presented in Figure S6 and Video S3 in the Supporting Information. Compared to the Janus drop impact (*µ*_h_/*µ*_l_ ≈ 545) in Figure 1d, when *µ*_h_/*µ*_l_ decreases to 112, the water part is more inclined to spread out in a semicircle, showing a smaller open angle. However, when the *µ*_h_/*µ*_l_ increases up to 1491, the glycerin part spreads out smoothly, and the water part fans out with a larger open angle (Figure S7, Supporting Information). Moreover, the number of daughter droplets emitted from the rim of the water part also increases with increasing *µ*_h_/*µ*_l_. Therefore, it can be demonstrated that an increase in the viscosity ratio of the Janus drop promotes a splash compared with the single‐phase water drop impact.

To obtain a quantitative understanding, with a large set of experiments, we can summarize the overall impact outcome in a phase diagram: a regime of the impact velocity (*v*) as a function of the viscosity ratio (*µ*_h_/*µ*_l_) of the drops (**Figure** **2**a). It is known that with increasing impact energy, the inertia of the expanding sheet may conquer both the surface tension and viscosity; thus, splashing can occur.^3^ As shown in Figure 2a, splash occurs only when the impact velocity reaches to a critical value for both the single‐phase water drop and the Janus drop. For example, a transition can be identified at approximately v ≈2.4, 2.2, 2.0, and 1.9 m s^−1^ for the single water drop and the Janus drop of $\mu_{\text{h}}\text{/}\mu_{\text{l}}\approx \;112,\;545$, and 1491, respectively. The corresponding Weber number for water *We*_w_ = *ρv* ^2^ *D*/σ is ≈188, 164, 133, and 119 and Reynolds number (*Re*_w_ = *ρvD*/*µ*) is ≈2851, 2660, 2392, and 2273, respectively (Figure S8, Supporting Information). Here ρ is liquid density, *D* is the drop diamter and *µ* is the liquid viscosity. It can also be found that the water part of the Janus drop is more likely to splash as the *µ*_h_/*µ*_l_ increases (Figure 2b). Therefore, it seems reasonable to conclude that the presence of glycerin can still trigger secondary droplets even when the impact is below the splash limit.

**Figure 2:**
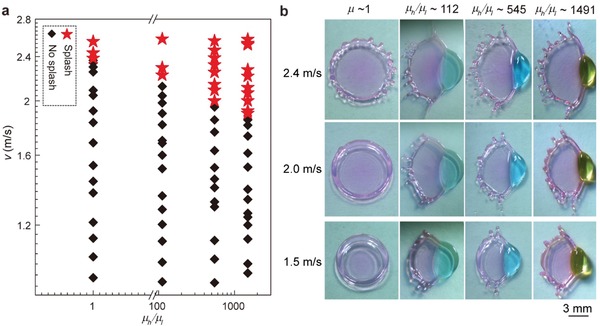
Control over the viscosity ratio and the results of splashing dynamics. a) Regime map of the outcome of impacting velocity *v* as a function of *µ*_h_/*µ*_l_. b) The maximum spreading phenomenon for the drop with different viscosity ratios at ≈1.5, ≈2.0, and ≈2.4 m s^−1^, respectively.

We further investigate how the viscosity ratio plays a role in the spreading stage. With increasing viscosity ratio, both the velocity of the moving drop and the reached maximum spreading diameter increase. By increasing the impacting kinetic energy, i.e., increase the impact velocity, the viscosity ratio effect can be further enhanced (**Figure** **3**a). However, the spreading of the glycerin part is suppressed (Figure 3b). Due to the special morphology of the shell‐like spreading, we quantify the expanding phase by measuring normalized spreading areas (Figure 3c,d). For pure water drop impact, a scaling law behavior $A_{\text{max}-\text{w}}\text{/}A_{0-\text{w}}\approx \;v$, was identified with $R_{\text{max}-\text{w}}\text{/}R_{0-\text{w}}\approx \;We^{0.25}$ in a previous report,^25^ is indeed observed at *v* values ranging from 0.95 to 2.5 m s^−1^, where $A_{\text{max}-\text{w}}=\;\pi R_{\text{max}-w}^{2}$, $A_{0-\text{w}}=\;\pi R_{0-w}^{2}$. However, for Janus drop, when *A*_max−w_/*A*_0−w_ is described by $A_{\text{max}-\text{w}}\text{/}A_{0-\text{w}}\approx \;v^{\alpha }$, α > 1; the value of α increases with an increase in the viscosity ratio *µ*_h_/*µ*_l_, where $\alpha (\mu_{\text{h}}\text{/}\mu_{\text{l}}:1491)\;\approx \;1.3>\alpha \left(\mu_{\text{h}}\text{/}\mu_{\text{l}}:545\right)\;\approx \;1.2>\alpha \left(\mu_{\text{h}}\text{/}\mu_{\text{l}}:112\right)\;\approx \;1.1$. Additionally, we found that the maximum spreading area of glycerin *A*_max−g_/*A*_0−g_ can also be described by a power law of $A_{\text{max}-\text{g}}\text{/}A_{0-\text{g}}\approx v^{\beta }$. For a single‐phase glycerin drop, the exponent β ≈ 0.5 is observed in Figure 3d, which is consistent with the previous report.^26^ However, for the glycerin part of the Janus drop, β < 0.5, and $\beta \left(\mu_{\text{h}}\text{/}\mu_{\text{l}}:1491\right)\;\approx \;0.25<\beta \left(\mu_{\text{h}}\text{/}\mu_{\text{l}}:545\right)\;\approx \;0.34<\beta \left(\mu_{\text{h}}\text{/}\mu_{\text{l}}:112\right)\;\approx \;0.4$. These results clearly demonstrate that the spreading of the water part is enhanced, while the glycerin part is suppressed. More remarkably, the effect of promotion or inhibition increases with an increasing viscosity ratio and impact velocity.

**Figure 3:**
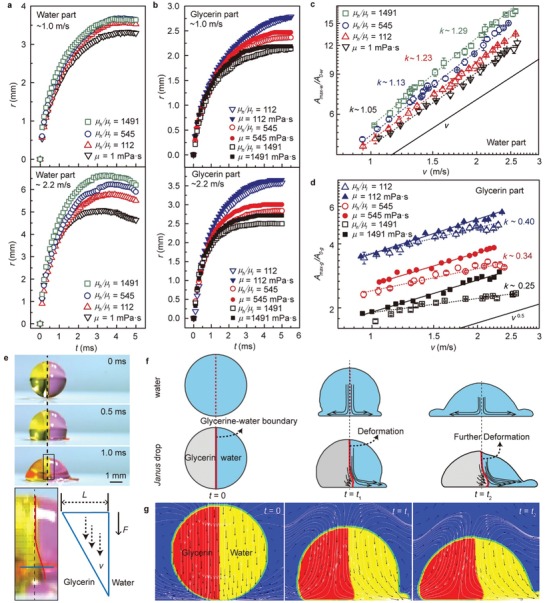
Mechanism and theoretical model of the splash promotion for Janus drop. a) Timecourse change in spreading radius *r* of the water part for the Janus drop at different impacting velocity. b) Timecourse change in spreading radius *r* of the glycerin part for the Janus drop at different impacting velocity. c,d) The normalized maximum spreading areas of the water part (*A*_max−w_/*A*_0−w_) and the glycerin part (*A*_max−g_/*A*_0−g_) as a function of impact velocity (*v*). e) Sideviews of the Janus drop (*µ*_h_/*µ*_l _ ≈ 1491) impact dynamics at *v* ≈1.2 m s^−1^. The inset shows the details of glycerin–water boundary. The blue line denotes the section of liquid. f) Schematic graph of liquid motion during drop impingement. The solid black arrows represent the direction of the flow field at this point. g) Flow patterns of Janus drop impact.

According to the above results and analysis, there must be an interplay between the glycerin and water during impact. From the side views of the impact, deformation occurs at the water/glycerin interface (Figure 3e). For a single‐phase water drop impact, the internal flow field is symmetrically distributed around the drop (the first row of Figure 3f). While for Janus drop, due to the non‐uniformed velocity field and liquid property, an asymmetric flow pattern is exhibited (the second row of Figure 3f). This effect is because when the Janus drop lands on the surface, the velocity of the glycerin part undergoes a sharp decrease due to the viscous dissipation, while the water part still expands at a certain velocity. Therefore, the shear stress *F* at the water/glycerin interface must be taken into account, which deforms the interface. Following the classical Newton's law of viscosity^27^

(1) $$\frac{F}{A}\approx \mu_{\text{h}}\frac{v}{L}$$

where *A* is the interfacial area between two components scaling as $\pi R_{0}^{2}$, *v* is the spreading speed, and *L* is the thickness of the flow, which means the offset distance of glycerol to water under the shear force of water, as shown in Figure 3e. For drop impingement, the stress *F* takes the inertia force of *ma*, where *m* is the mass of the drop, *a* ≈ *v* ^2^/*R*_max_ is the acceleration of the interface, as estimated in the previous report.^22^ Since the droplet size is smaller than the capillary length, the gravity force can be completely neglected. Substituting in all these terms to Equation (1) results in the relation of offset distance

(2) $$L\approx \mu \frac{\pi R_{0}^{3}}{\text{m}\sqrt{v}}$$

Apparently, there is a linear correlation between the *L* and *µ*. That is, the higher the viscosity of the glycerin part is, the stronger the deformation at the water/glycerin interface is. Such an offset of the glycerin occupies the spreading space of the water part near the solid–liquid surface and forms a glycerin incline part. As a consequence, it is easier for the upper fluid flowing toward to the water part in *x*‐axial direction and accelerating the expansion.

To confirm the above analysis, numerical simulations were conducted using the commercial software ANSYS Fluent (Version 5.6), where the volume of fluid (VOF) method was employed to trace the interfaces (see details in the Experimental Section and Figure S9, Supporting Information). Figure 3g shows the flow patterns in the early spreading stage of the Janus drop $\left(\mu_{\text{h}}\text{/}\mu_{\text{l}}\approx \;1491\right)$ after impact. Good agreement between the numerical results and our schematically models (Figure 3f) can be found. From the diagram of streamlines and velocity, we also find that the stagnation point shifts from the initial impact point to the glycerin side, in accordance with our experiments (Figure S10 and Video S4, Supporting Information). Such a phenomenon further proves the existence of shear force at the two‐phase interface. Based on the analyses and simulation results, one can regulate the spreading promotion by controlling the viscosity ratio (*µ*_h_/*µ*_l_), which is consistent with our experimental results.

Researchers have extensively studied the fingering instability on spreading rim during splashing.^22^ In our experiment, we also observed a feature similar to fingering around the rim as shown in the inset of **Figure** **4**. Due to the special morphology of the spreading water, we focus on the behavior and the number of corrugations (*N*) per unit arc length (*L*) at the maximum spreading point under different impact velocities. As the viscosity ratio increases, the drop exhibits higher rim instability. This behavior can be explained by the R‐T instability, which is first proposed by Allen.^22^ This fingering instability occurs at the interface of two fluids with different densities when the acceleration *a* points from the light fluid ρ_1_ toward the heavy fluids ρ_2_. For a fast‐growing wavelength, a linear analysis of the RT instability provides the following expression^22^

(3) $$\frac{1}{\lambda }=\frac{1}{2\pi }\sqrt{\frac{a\left(\rho_{2}-\rho_{1}\right)}{3\gamma }}$$

where λ = *L*/*N* is the wavelength, *a* ≈ *v* ^2^/*R*_max_ is the acceleration of the interface, and γ is the surface tension of the liquid. Substituting all terms into Equation (3), we can obtain the expression of *N*/*L*

(4) $$\frac{N}{L}=\frac{1}{\lambda }\;\approx \;\frac{1}{A_{\text{max}}^{0.25}}v$$

where *A*_max_ is estimated to be $\pi R_{\text{max}}^{2}$. That is, as the spreading velocity increases, there will be more fingers forming on the periphery of the water drop (Figure 4), where the spreading velocity can be increased either by increasing the impact velocity or add a higher viscous liquid part. To explore the effects of Janus drop size on the impact results, we conducted experiments by reducing the size of the Janus drop (*µ*_h_/*µ*_l_ ≈ 1491) from 3.1 to 2.7 mm in diameter. The results demonstrate that the drop size would not influence the rim instability, as shown in Figure 4.

**Figure 4:**
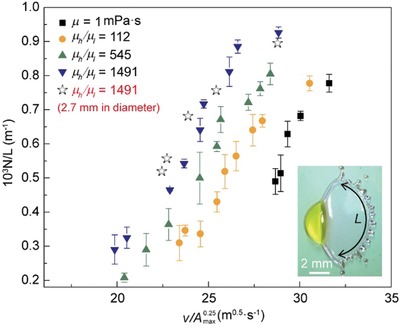
The number of corrugations *N* per unit arc length *L* at the maximum spreading as a function of $\frac{1}{A_{\text{max}}^{0.25}}v$. The gray stars represent the results of 2.7 mm‐diameter Janus drops.

When the viscosity ratio and impact velocity are sufficiently high, the water part can be almost completely separated from the glycerin part of the Janus drop. As shown in **Figure** **5**, the Janus drop with $\mu_{\text{h}}\text{/}\mu_{\text{l}}\approx \;1491$ splits into two parts during the retraction phase when the velocity exceeds a ctritical value of ≈1.85m s^−1^(Figure S11 and Video S5, Supporting Information). We attribute this phenomenon to the high viscosity ratio of the two compound, the superhydrophobicity of the substrate, and the high kinetic energy of the recoiling in the water part. Due to the high difference in viscosity, the water part could fully spread and then retract with velocity roughly from three directions (arrows in Figure 5), while the kinetic energy of the glycerin part was significantly dissipated, and thus this part slightly deformed and pinned on the surface. The superhydrophobicity of the substrate then facilitated the strong retraction of the water part, which had the only constraint of glycerin part and finally contracted into a horizontal ‘V' shape (e.g., images at 9.0 ms shown in Figure 5). Although the glycerin and the water parts were still connected at this stage, the rather high kinetic energy of the water part further seperated it from the viscous glyceirn, resulting in two separate entities. When the viscosity ratio and impact velocity are sufficiently high, the water part can be almost completely separated from the glycerin part of the Janus drop. According to the result of our experiments, Janus drop splitting only occurs at the viscosity ratio of ≈1491 when the impacting velocity exceeded ≈1.85 m s^−1^ (Figure S11 and Video S5, Supporting Information). For Janus drop with $\mu_{\text{h}}\text{/}\mu_{\text{l}}\leq \;545$, no splitting was observed, probably caused by the strong adhesion.

**Figure 5:**
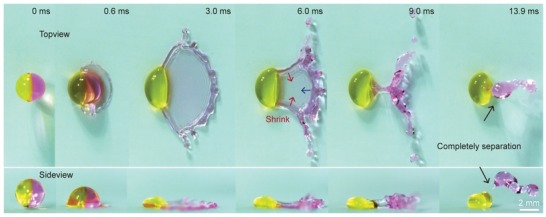
The drop splitting phenomenon of Janus drop (*µ*_h_/*µ*_l _ ≈ 1491) at the impact velocity of ≈1.85 m s^−1^.

In summary, we have demonstrated a novel impact phenomenon that promotes splashing and instability of a low‐viscosity drop by imposing a highly viscous drop. Distinct from single‐phase drop impacts, the impinging Janus drop shows an asymmetric shape throughout the impact process due to the different spreading and retracting dynamics. Along with the theoretical analysis and simulation, we show that the shear force induced interface deformation to understand the splashing promotion. Notably, instead of preventing splash phenomenon in previous studies,[qv: 9,16b] our work may have broad applicability in processes that accelerate the splashing of drops. We envision that our discovery can be extended to applications where splash with good control is advantageous including ink‐jet printing,^8^ pesticide deposition,^9^ and spray cooling process.^10^

## Experimental Section

*Materials and Fabrication of Superamphiphobic Surface*: Glycerin (for molecular biology, ≥99%), rhodamine B, and methyl blue were purchased from Sigma‐Aldrich, tartrazine and fast green FCF from Aladdin. All chemicals and solvents were used without further purification. Glass slides used as substrates were sonicated, rinsed with Milli‐Q water and dried with nitrogen. Superamphiphobic surfaces were fabricated on glass base by a candle soot‐templated structure with silica shell followed by fluorination as the method in the previous report. Further fabrication details are given in Deng et al.^24^

*Characterization*: The morphology of the nanostructure was characterized by scanning electron microscopy (FEI Inspect F). The impact dynamics of drops were recorded by high‐ speed cameras. The impact velocity (error < 5%) of the drop was also determined from these movies and processed using a custom‐programmed MATLAB (Math_Works Inc., USA) algorithm.

*Janus Drop Formation and Drop Impact Process*: The generating process of Janus drop was straight. First, a reproducible water drop (*R*_l_ ≈ 1.25 mm) was suspended on syringe 1; then, a glycerin drop (*R*_h_ ≈ 1.25 mm) was squeezed out of syringe 2 and coalesced with the water drop, thus forming a Janus drop $\left(R\;={\left(R_{\text{l}}^{3}+R_{\text{h}}^{3}\right)}^{1/3}\;\approx \;1.55\;\text{mm}\right)$ which presented a clear glycerin–water boundary due to the mortal viscosity discrepancy. The resulted Janus drop spins slowly as it moves downward. The rotation trajectory was obtained by measuring the variation of *x* and *y* from *O* to *Z* in view of the point *A* when the drop was released from the height of 5 cm, as shown in Figure S1a in the Supporting Information. The displacement component *x* was much less than the displacement component *y* at the same time scale (Figure S1b, Supporting Information). It could be obtained that the rotation speed, which approached 0.02 m s^−1^, was far less than the impacting velocity which is up to 1 m s^−1^. Therefore, the effect of rotation was ignored in the impacting experiments. The surface tension in these mixtures did not vary much and remained between 72 mN m^−1^ (pure water) and 64 mN m^−1^ (glycerin).

A series of Janus drops were created with different viscosity ratios by changing the viscosity of the viscous part using the same method aforementioned. The different viscosity of the glycerin parts (e.g., ≈112 and 545 mPa · s) are obtained by water–glycerin mixtures at different ratios (corresponding to 85 and 95 wt%), spanning the whole range from pure water (≈10^−3^ Pa · s) to pure glycerin (≈1.4 Pa · s). Note that the order of magnitude of the diffusion coefficient for the binary system glycerol‐water is on the order of 10^−10^ m^2^ s^−1^.^28^ Therefore it could be estimated that the thickness of the diffusion layer at the glycerin–water boundary was several tens of micrometers, which was very small during the drop impacting process (a few microseconds). To better disgusting these Janus drops, the water, 100 wt% glycerin, 95 wt% glycerin, and 85 wt% glycerin are dyed by rhodamine B, lemon yellow, methyl blue, and fast green FCF, respectively. The liquids properties used in the experiments are summarized in Table S1 in the Supporting Information.

All impacting drops were released from heights between 5 and 30 cm. Two high‐speed cameras (Photron SA5 and Photron SA‐Z, Japan) were used to record the impacting dynamics from the top and side views concurrently at frames per second.

## Conflict of Interest

The authors declare no conflict of interest.

## Supporting information

Supporting Information

Supplemental Video 1

Supplemental Video 2

Supplemental Video 3

Supplemental Video 4

Supplemental Video 5
