# Supplementary material for: Prompting Splash Impact on Superamphiphobic Surfaces by Imposing a Viscous Part
Source: Adv Sci (Weinh). 2020 Jan 10;7(4):1902687. doi: 10.1002/advs.201902687 (PMC7029656; doi:10.1002/advs.201902687)
Supplement: Supplementary file 1 — Supporting Information [file ADVS-7-1902687-s001.pdf]

Copyright WILEY-VCH Verlag GmbH & Co. KGaA, 69469 Weinheim, Germany, 2020.

ADVANCED SCIENCE
Open Access

Supporting Information

for *Adv. Sci.,* DOI: 10.1002/advs.201902687

Prompting Splash Impact on Superamphiphobic Surfaces by Imposing a Viscous Part

Fanfei Yu, Shiji Lin, Jinlong Yang, Yue Fan, Dehui Wang, Longquan Chen, and Xu Deng**

Supporting Information

Prompting Splash Impact on Superamphiphobic Surfaces by Imposing a Viscous Part

Fanfei Yu, Shiji Lin, Jinglong Yang, Yue Fan, Dehui Wang, Longquan Chen, and Xu Deng**

Contents

Supplementary Videos 1-5

Supplementary Figures S1-11

Supplementary Table

Supplementary References

Supplementary Videos

Video 1. The formation process of *Janus* drop.

Video 2. The generation of prompt splash by *Janus* drop ($\mu_\mathrm{h}/\mu_\mathrm{l}{\sim}545$) impacting, compared with the event of single-phase water drop impacting at the impact velocity of $\sim 2.2\ \mathrm{m/s}$.

Video 3. The impact dynamics of *Janus* drop with different viscous ratio ($\mu_\mathrm{h}/\mu_\mathrm{l}{\sim}112$ and 1491) at the impact velocity of $\sim 2.2\ \mathrm{m/s}$.

Video 4. The bottom-view impacting dynamics of the *Janus* drop ($\mu_\mathrm{h}/\mu_\mathrm{l}{\sim}1491$) at $\sim 1.25\ \mathrm{m/s}$ on a hydrophobic glass surface.

Video 5. *Janus* drop splitting. The water part can be separated from the glycerin part for a *Janus* drop with $\mu_\mathrm{h}/\mu_\mathrm{l}{\sim}\ 1491$ when the impacting velocity exceeds $\sim 1.85\ \mathrm{m/s}$.

Supplementary Figures

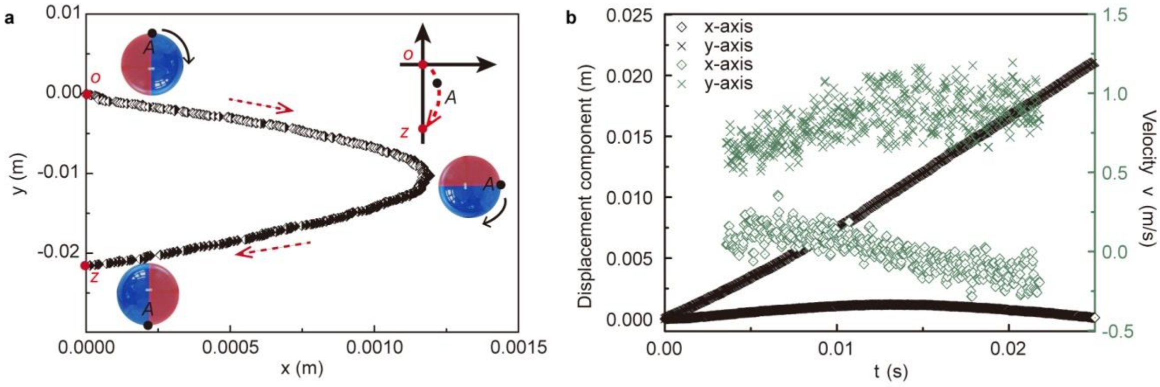

Figure S1. Influence of slight rotation on the impacting velocity during the *Janus* drop generating process. a) The variation of *x* and *y* from *O* to *Z* in view of the point *A* at the edge of *Janus* drop. b) The displacement component and velocity of point *A* in *x*-axis and *y*-axis.

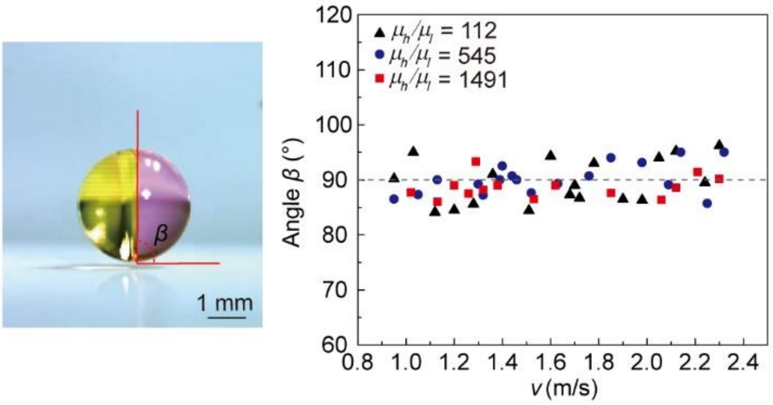

Figure S2. Error analysis of the angle between the glycerin-water boundary and the horizontal surface.

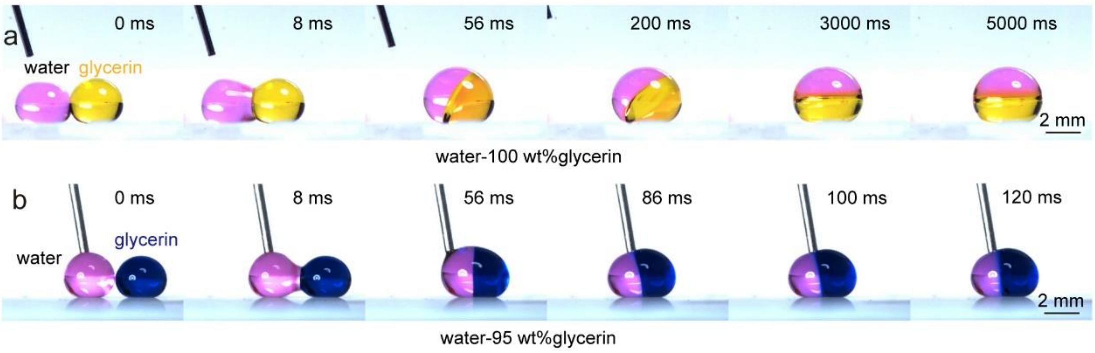

Figure S3. Timescale for maintaining the clear glycerin-water boundary at different viscosity ratios.

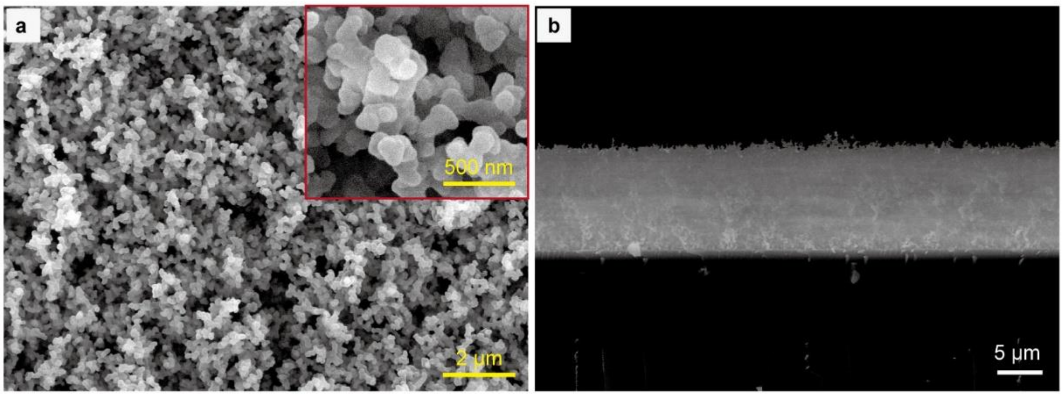

Figure S4. The structural morphology of the superamphiphobic surface. SEM images shows the a) topview and b) sideview of the micro/nano-structure of the substrate. The inset (red box) shows the magnified image on a particular area of a).

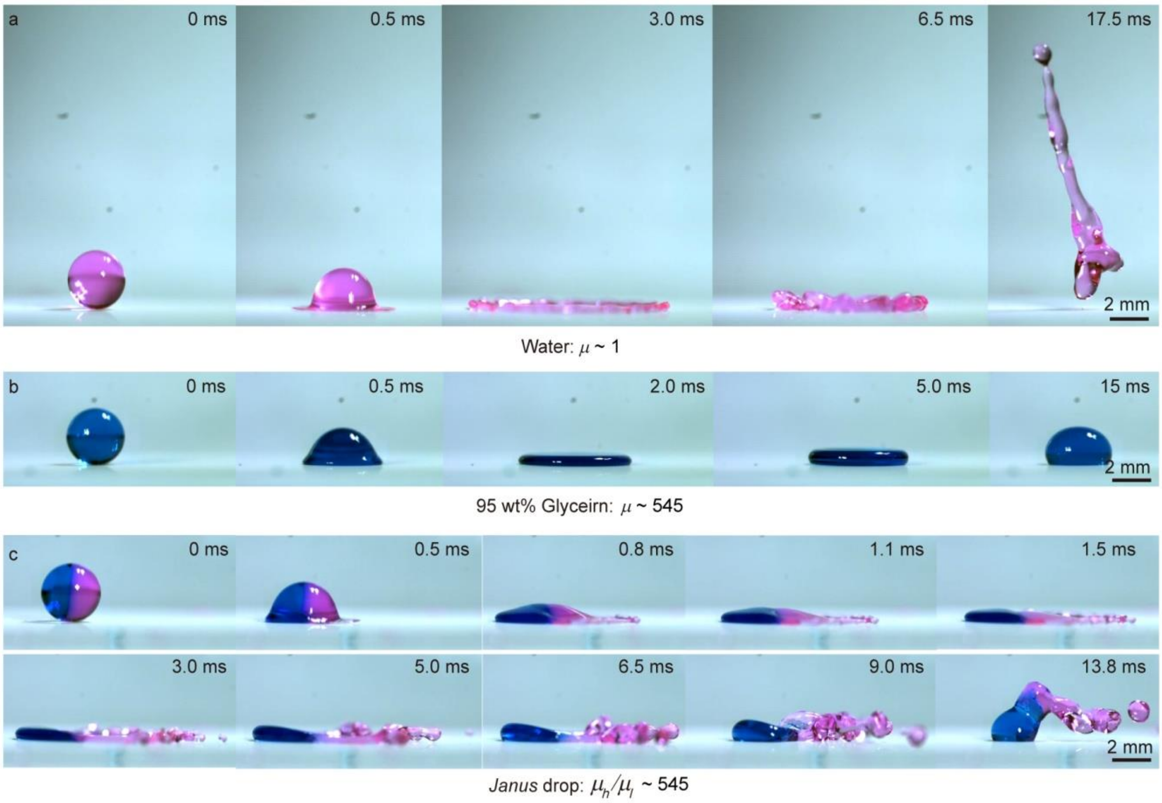

Figure S5. Impacting dynamics of the single-phase drop and *Janus* drop (Sideviews of Figure 1b-c). Side-view images of impacting dynamics at the velocity v~2.2 m/s for a) single-phase water drop (μ~1 mPa · s), b) single-phase 95 wt% glycerin drop (μ~545 mPa · s), and c) *Janus* drop ($\mu_\mathrm{h}/\mu_\mathrm{l}$~545).

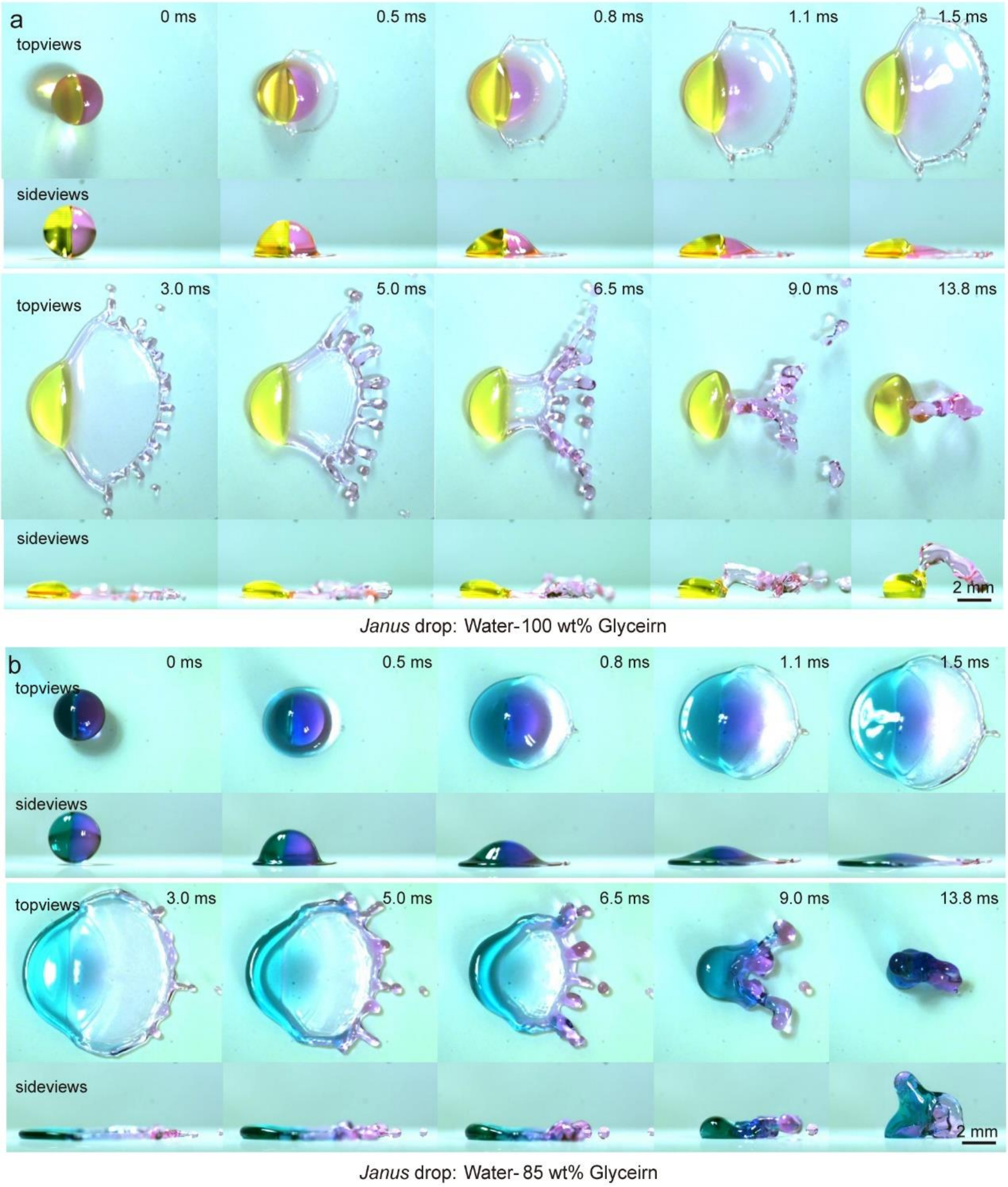

Figure S6. Impacting dynamics of the *Janus* drop with different viscosity ratios. Top and side-view images of impacting dynamics of impact dynamics at the velocity $v{\sim}2.2\ \mathrm{m/s}$ for a) *Janus* drop ($\mu_\mathrm{h}/\mu_\mathrm{l}{\sim}1491$), b) *Janus* drop ($\mu_\mathrm{h}/\mu_\mathrm{l}{\sim}112$).

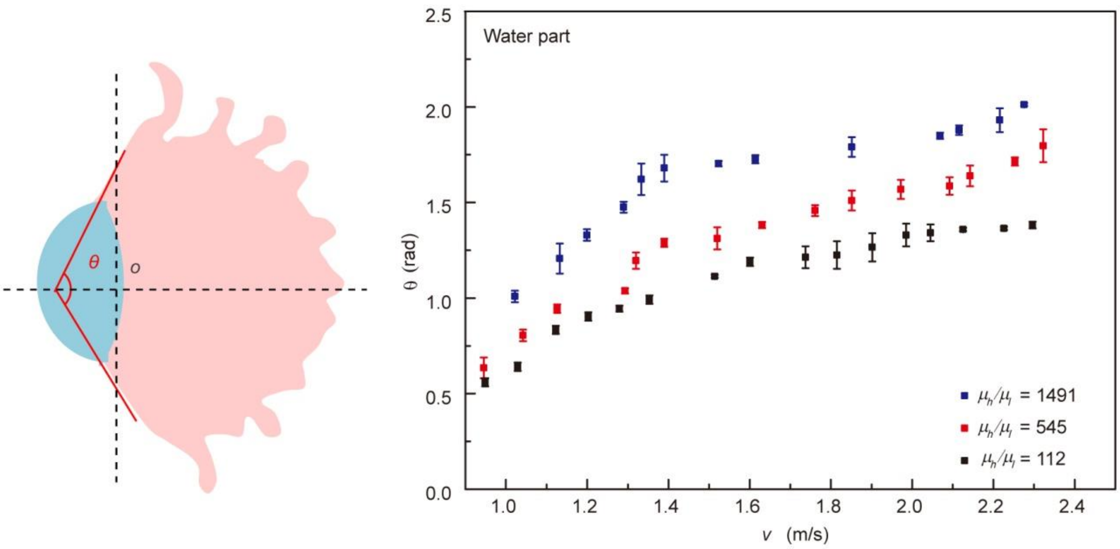

Figure S7. Spreading open angle of the water part for *Janus* drop. The angles (θ) between the two sides of the water part at the maximum spreading phase as a function of impact velocity.

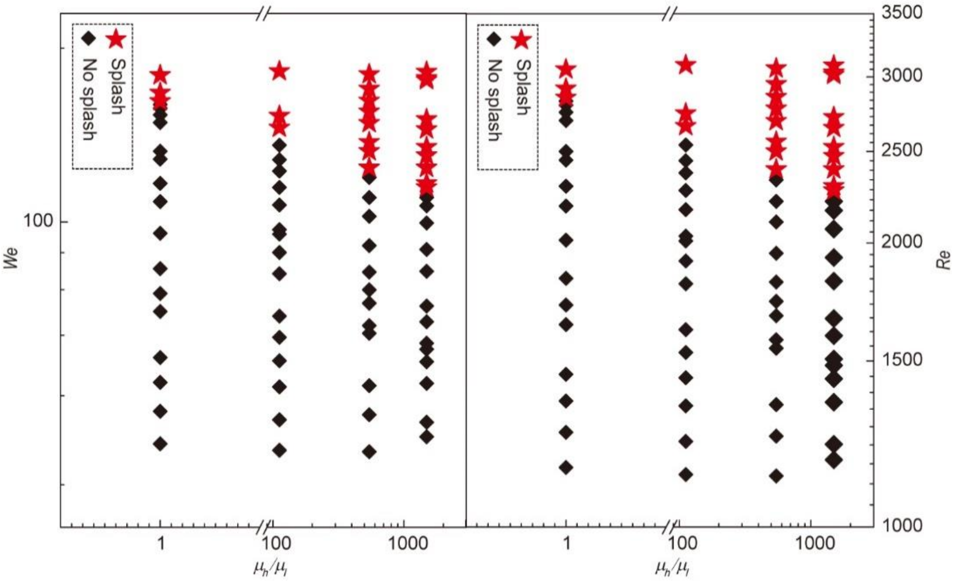

Figure S8. Impact phase diagrams as a function of the $We_{\mathrm{w}}/Re_{\mathrm{w}}$ and the viscosity ratio of the *Janus* drop.

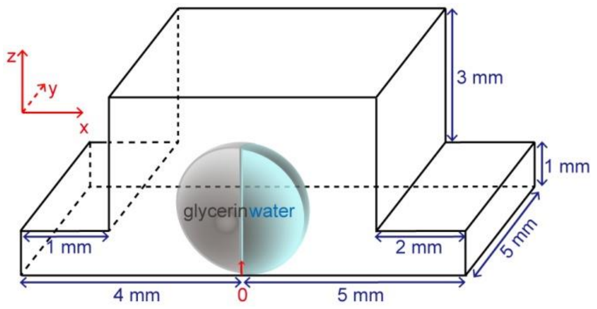

Figure S9. Simulation of the *Janus* drop impact. The Simulation was conducted by a commercial software ANSYS Fluent (Version 5.6) by VOF methods. A 3D trapezoid grids containing $\sim 1.84\mathrm{e}+5$ rectangular grids was adopted in the simulation of the *Janus* drop ($\mu_{\mathrm{h}}/\mu_{\mathrm{l}} \sim 1491$) impact (Figure S6).The near-wall grid is densified self-adaptively to enhance the calculation precision. The corresponding time step, $2.0\mathrm{e}-6$ s, is effective and convergent. The wall is defined as the non-slipping boundary. The contact angle between the wall and the water part (or glycerin part) is 150°. The surface tension of water and glycerin are 72.8 and $63.0\ \mathrm{mN/m}$. The other boundaries are pressure-outlet or pressure-inlet. The coupling equations of pressure and velocities were solved using the PISO (Pressure Implicit Split Operator) method.

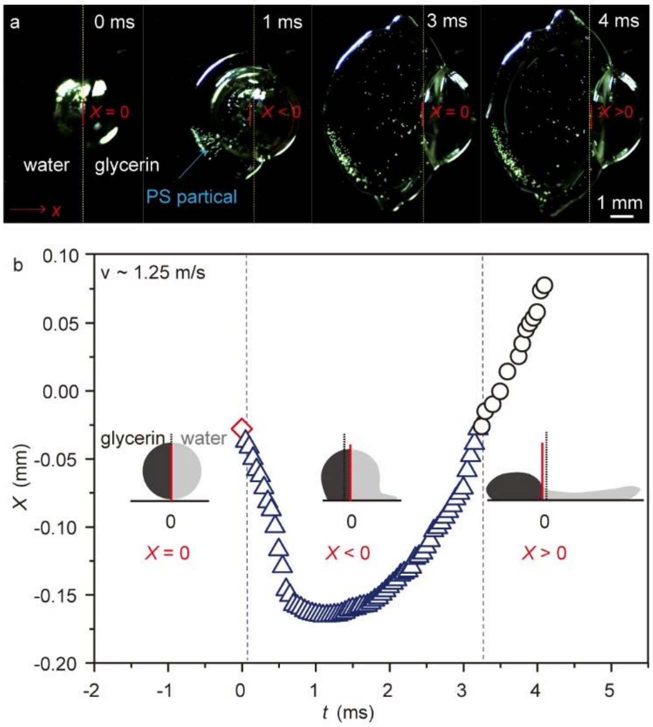

Figure S10. Measurement of the stagnation point motion. a) To better track the changes of stagnation point, we record the *Janus* drop ($\mu_{\mathrm{h}}/\mu_{\mathrm{l}}$~1491) impact dynamics from the bottom view by using a transparent hydrophobic glass surface at a velocity v~1.25 m/s. The water part was added with some PS particals (10 µm in diameter) for visuality. b) The stagnation point changes over time.

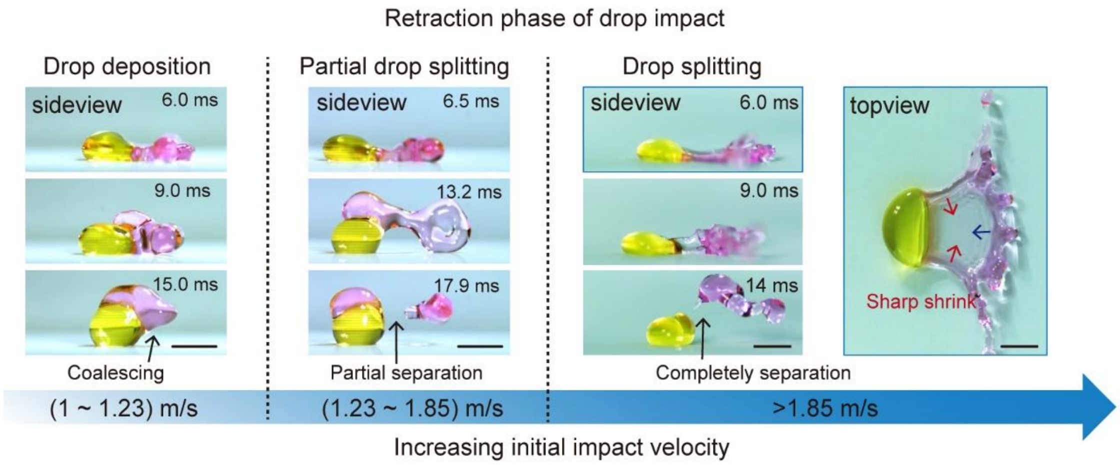

Figure S11. The retraction phase and drop splitting phenomenon during drop impacting process.

Supplementary Table

Liquid (20 ℃)	Radius (mm)	ρ (kg m^{-3})	σ (mN m^{-1})	μ (mPa·s)
water	1.55	997	72	1
85 wt% glycerin	1.55	1217	64	112
95 wt% glycerin	1.55	1284	64	545
100 wt% glycerin	1.55	1261	63	1491

Table S1. A summary of the liquid properties. Experimental parameters and liquid properties of drops[1-3].

References

[1] M. L. Sheely, *Ind. Eng. Chem.* **1932**, 24, 1060.

[2] L. W. Bosart, A. O. Snoddy, *Ind. Eng. Chem.* **1928**, 20, 1377.

[3] C. M. Romero, M. S. Paéz, *Physics and Chemistry of Liquids* **2006**, 44, 61.
